# Validation of a roadmap for mainstreaming nutrition-sensitive interventions at state level in Nigeria

**DOI:** 10.1186/s12937-020-00612-1

**Published:** 2020-09-09

**Authors:** Oluchi Ezekannagha, Scott Drimie, Dieter von Fintel, Busie Maziya-Dixon, Xikombiso Mbhenyane

**Affiliations:** 1 Division of Human Nutrition, Faculty of Medicine and Health Science, Stellenbosch University, Cape Town, South Africa; 2 International Institute of Tropical Agriculture, Ibadan, Nigeria; 3 Department of Economics, Stellenbosch University, Stellenbosch, South Africa

**Keywords:** Nutrition-sensitivity, Operational validation, Nigeria, Intervention modification, Roadmap development

## Abstract

**Background:**

National programs are often developed with little consideration to the sub-national local factors that might affect program success. These factors include political support, capacity for implementation of program and variation in malnutrition indices being tackled. State context factors are evident in the distribution of malnutrition (e.g. high prevalence or gap among Local Government Areas), in the implementation of nutrition-sensitive interventions (e.g. access to early childhood education) and in the political economic context (e.g. presence of external funding agencies). Context is shaped by the economy, population, religion, and poverty, which impact everyday lives. Considering these contexts, a roadmap was developed and validated. The aim of this paper is to report expert review and stakeholder validation to determine feasibility of the developed contextualised roadmap for two Nigerian states.

**Methods:**

A validation tool was developed and reviewed using three experts. The content review occurred in two rounds to obtain recommendation and revisions of the developed roadmap and the validation tool. A pilot test of the roadmap and validation tool was done using two stakeholders in South Africa. The roadmap and the validation tool were then sent to the stakeholders and potential end-users in Nigeria using electronic media. Two research assistants were also engaged to deliver and collect hard copies to those who preferred it.

**Results:**

Of the ten stakeholders invited, nine responded. All participants showed an adequate understanding of the roadmap as evidenced by the scores given. Responses regarding the translation of the roadmap to implementation varied. The majority (86,6%) either strongly agreed or agreed that the actions were translatable (43.0 and 43.6% respectively).

**Conclusions:**

The final roadmap comprises of actions that are appropriate for the state’s context. It is recommended that stakeholders or end-users of any programme must be involved in the validation of such contextual programmes to improve chances of success.

## Introduction

Child malnutrition remains high among under-fives in Nigeria, despite programming and policy interventions. The country-level stunting rate of 37% [1] is high according to World Health Organisation (WHO) malnutrition prevalence severity [2]. The variation in malnutrition prevalence in Nigerian States makes it difficult to replicate programs without adaptation. The two states (Anambra and Kebbi) used in the study have a stunting prevalence of 18.4 and 60.6%, respectively.

Successful approaches to address malnutrition need the input and actions of multiple sectors and stakeholders as indicated by the United Nations Children’s Fund (UNICEF) conceptual framework [3]. Factors within the different causal levels of the UNICEF framework of nutrition exert influence on under-fives malnutrition and health outcomes. The influence of the underlying and basic causal levels on malnutrition reduction have not been fully maximised [4, 5].

Context as a term is broad, with meanings and implications cutting across epistemologies [6] engaging from aetiology, efficacy, effectiveness to study settings as it applies in public health. Luoto et al. has reported on the limitations to reporting context in studies, with subsequent challenges during implementation of interventions. This article focuses on importance of context both for the replicability and scalability of interventions. Replicability is the dissemination of interventions without further adaptation while scalability is an increased reach of an intervention [7]. Varying political, personnel, population characteristics and infrastructural factors affect scalability [5]. This could also be hypothesised to influence replicability.

The field of nutrition interventions are notable for effectiveness studies [8, 9]. Many interventions are not scalable, given the level of resources they require. It is problematic to simply scale-up or replicate even the most efficacious and effective interventions. This increases the need for testing and adaptation to account for complex environments [10]. Implementation context does not include the intervention characteristics alone but also draws from the implementing environment such as leadership and communication strategies. Accounting for some or all these variables ensure that interventions have higher chances of success.

These variables are higher when the programs are being implemented at a large scale by governments. Indeed, one of the importance of implementation science is the realization that well-thought-out interventions become ineffective when implemented in the field, especially when government actions and bureaucracies come into play [11–13].

Although, efforts have been made to ensure that interventions in the nutrition-sensitive sectors are aimed at impacting overall nutrition [5], achieving mainstreaming of such efforts have not occurred even when suggestions on indicators and pathways have been provided [14, 15]. Nutrition-sensitive interventions is defined by Ruel and Alderman [5] as actions, policies or programmes that address the underlying determinants of malnutrition by incorporating specific nutrition goals and actions. Efforts on mainstreaming nutrition into wide coverage programs implemented by governments have been missing. If efforts are not made to make nutrition mainstreaming a part of governments programming, programs with wide coverage risk not being nutrition-sensitivity. Literature reveals an enormous interest by researchers on nutrition-sensitivity of cross-cutting sectors. However, literature has not been clear on contextual recommendations or interventions that best fit regional or national operational and epidemiology realities. The obvious lack of research on contextual interventions supports the need to develop evidence-guided and implementer-validated roadmap on mainstreaming nutrition into nutrition-sensitive sectors.

The paper aims to report on validation of a roadmap for mainstreaming nutrition into nutrition-sensitive sectors in Kebbi and Anambra States in Nigeria. Specifically, the validation sought to explore if the developed roadmap will function as intended once placed in the stakeholder’s environments and to assess the roadmap’s likelihood for success in mainstreaming nutrition initiatives in the states in Nigeria. The validation sought to explore specific themes on understanding, translation, acceptability, demand for the roadmap, implementation, practicability, and feasibility, integration, and political buy-in. For researchers and implementers, the outcomes of this study can provide structure and template for providing detailed guidelines to any unit of government – local gorvenment area (LGA), state, regional or national on specificity regarding nutrition-sensitive mainstreaming.

This paper forms part of a larger study that employed the Mainstreaming Nutrition Initiative Assessment (MNIA) Framework by Menon et al. [16] and a mixed method approach with the aim of developing a roadmap for mainstreaming nutrition into nutrition-sensitive sectors at the state level in Nigeria.

### Preceding phases: domain assessment and results

The study began with a quantitative arm (Phase 1) that used Small Area Estimation (SAE) methods [17] to estimate LGA prevalence of stunting in Nigeria. This was followed by exploration of the socio-political context using in-depth interviews of stakeholders and site visits to the interventions by the researcher to engage with the implementers and the beneficiaries of one programme in each ministry (Agricultural Transformation Agenda Support Programme 1 (ATASP-1), Early Childhood Development (ECD) Education, Environmental Sanitation, and Skills Acquisition).

Phase 3 aimed to explore political commitment analysis in the context and awareness of MNIA framework dynamics in the identified states. In Phase 4, the data and information from the previous phases were employed to develop a roadmap for mainstreaming nutrition-sensitivity in both states, which was also validated by the stakeholders. This article aims to report on the validation of the contextual roadmaps.

## Methods

### Research design

This phase of the study employed three steps; first two rounds of expert content review of developed roadmap and validation tool, second piloting of the roadmap and validation tool and finally stakeholder validation of roadmap using piloted validation tool.

### Participant selection

Purposive sampling was used to select three experts who were knowledgeable about the subject matter, willing to provide the information sought and participate in both rounds of the roadmap content review and validation tool development [18]. Experts were presented with the study aim and findings of the preceding phases as a background to the roadmap review to ensure relevance. The participants for the validation phase were three subject matter experts, two stakeholders from South Africa and nine Stakeholders for the two states in Nigeria. Both genders were represented in the three experts used and all had more than 10 years’ experience.

The roadmap and validation tool were first pilot tested among similar stakeholders identified in South Africa. Ten stakeholders were invited for pilot-testing but only two returned the validation tool and the roadmap. Two senior government officials from Nutrition/Agriculture in South Africa responded for piloting the validation tool. The same procedure that would be employed in validation were used in pilot-testing. The responses to the pilot-testing were incorporated and used to refine the instrument to validate the roadmap.

For stakeholder validation in Nigeria, purposive sampling was used to select a director from the relevant ministries, preferably one in charge of the process evaluation programme, for the operational validation of the roadmap and the State Nutrition Officer who serves as the secretary of the State Committee on Food and Nutrition. The target was 10 stakeholders per state representing Early Childhood Education, Agriculture, Environment, WASH, and Social Welfare ministries. A total of 9 stakeholders (5 in Anambra State and 4 in Kebbi State) completed and returned the validation questionnaire. The participants were middle-aged high-ranking civil servants working in various ministries at the study states in Nigeria. Four were males and five females; two were from Health/Nutrition, two from Agriculture, two from Environment, two from Social welfare and one from Education.

### Data collection

**Fig. 1:**
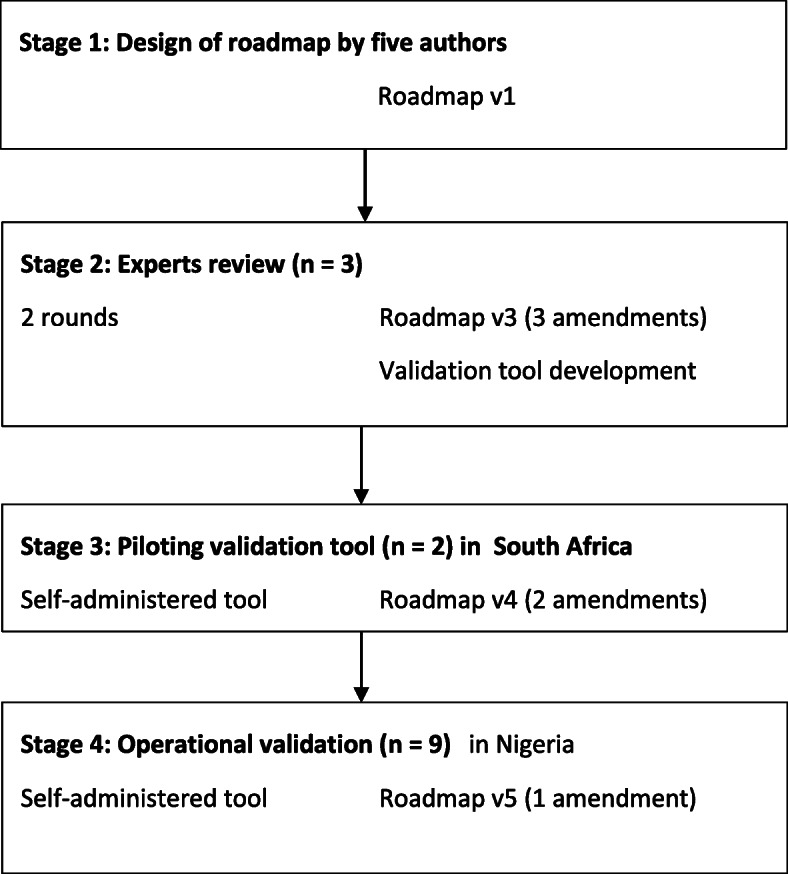
Summary of the Phase Four development stages for the development and validation of the roadmap

Data was collected in stages as depicted in Fig. 1.

### Initial roadmap draft

The roadmap development process applied the development stages of Whittaker et al [19]. The development follows five stages: normative, empirical, consensus, publishing, and implementation [19]. For this roadmap, only the first three stages were applicable, since the last two will happen in the long term.

The process of developing the roadmap took into consideration the findings from all three domains from phases 1 to 3. The nutrition-sensitive assessment identified the current and potential nutrition-sensitivity of programmes. This informed the key nutrition-sensitive principles that needed to be promoted by the ministries. The process evaluation was used to develop strategies for strengthening programme operation. The political economy analysis identified the pathway of nutrition advocacy. The utilisation of both the quantitative and qualitative methods described increased the data credibility, aiding the researchers in understanding the complexity of context-specific nutrition-sensitive mainstreaming in the states [20] and setting priorities that allow targeting through the established programme operations and pathway. The above processes are illustrated in Table 1.

**Table 1:** Methods and tools used in assessment of domains

Phase and methods	Selected findings
***Phase 1: Small Area Estimation of malnutrition***	
*Approach:* Quantitative *Aim:* To determine LGA prevalence’s of malnutrition in Anambra and Kebbi States. *Data collection strategy:* Use of secondary data and Small Area Estimation methods.	The study’s estimations data from this study, Osgood-Zimmerman et al. (2018) [22] and the Nigerian Demographic Health Survey was used in the development of the roadmap.
***Phase 2: Nutrition-sensitive and potential to be nutrition sensitive assessment***	
*Approach:* Qualitative *Aim:* To assess the operational realities in the nutrition-sensitive sectors and *process evaluation* *Data collection strategy:* Nutrition-sensitivity assessment; Potential to be nutrition sensitive assessment; Theory-based Process evaluation. *Data collection:* Document review and key informant interviews and site observation	Most programmes had good to excellent potentials to be nutrition sensitive. Inadequate implementation in most programmes and sectors, except agriculture. Numerous falter points in the programme impact pathways of the programmes. Strengthening of coordinating institutions and mechanisms.
***Phase 3: Political commitment assessment***	
*Approach:* Qualitative *Aim:* To assess the socio-political realities in nutrition-sensitive sectors. *Data collection strategy:* Two workshops administering the Political Commitment, Rapid Assessment Test [23].	Existing political commitment mainly for nutrition-specific interventions. Higher political commitment to nutrition in Kebbi than Anambra.
**Phase 4: Development and Validation of the roadmap (reported in this article)**	
*Approach*: Qualitative and quantitative *Aim:* To develop and validate a roadmap from mainstreaming nutrition in Anambra and Kebbi States. *Data collection strategy:* Merging data from the three phases to develop a detailed contextual roadmap and literature. Expert and stakeholder validation of developed roadmap.	Developed and validated roadmap

### Expert content review of the roadmap

Three experts were involved in the revision of the roadmap during development. Content reviews mainly hinged on literature and empirical findings. Major revision made at this stage was the inclusion of the indicators to the mainstreaming table and editing to make the roadmap concise. When no further changes were made and experts agreed on a version, the process was halted and taken to signify theoretical saturation [21]. The experts reworked the roadmap until version 3 and the validation tool were produced.

### Validation tool

The roadmap was validated by participants using the validation tool adapted from Bowen et al. [16]. The validation tool was developed by the researcher and the expert team based on the outcome and shape of the roadmap. This step becomes vital given that products and processes need validation when process output cannot be verified by implementing, monitoring, and evaluating an innovation. The validation tool measured the following constructs: understanding, translation, acceptability, demand, implementation, practicability, integration, and potential buy-in. There were statements linked to the constructs, and participants had to use the scale for scoring (Strongly agree [2); Agree [1]; Neutral [0]; Disagree [− 1]; Strongly disagree [− 2]. These were translated into percentage response when reporting. Table 2 gives an overview and content of the validation tool.

**Table 2:** Domains of the validation tool

Domain^a^	Description of the domain	Number of items assessed for domain ^b^
Understanding	Ease of roadmap and roadmap contents comprehension	4
Translation	Measures facilitators of translation such as appropriateness, context nuances and budget feasibility of the roadmap	4
Acceptability	Perception, satisfactoriness and organizational fit of the roadmap	5
Demand for a roadmap	Perceived existing demand and ability of roadmap to address existing gaps in programs	3
Implementation	Measures possible execution of all sections and actions listed in the roadmap	4
Practicality	The ability of existing financial and human resources to implement the roadmap	2
Integration	The roadmap’s ability to fit into the current programming sustainably	3
Political buy-in	The Ministry’s support for adoption of roadmap	1

^a^Domain is the main assessment areas

^b^Number of items assessed for domain are single items that measured in each domain

### Pre-testing of validation tool for the roadmap

The validation tool employed in the validation exercise was pilot-tested. Ten stakeholders were invited for pilot-testing with two returned responses. The validation tool and the roadmap were emailed to the invited participants, and they were requested to return the completed validation tool with additional comments. Several reminders were sent, and after 2 months it was decided that the inputs of the two were substantive enough to improve the tool. The main issues raised during pilot testing were recommendations regarding unclear validation questions and repetitive questions which required same response, and these were merged.

### Feasibility validation of the roadmap version 4

#### Validation methods

The operational validation attempted to verify key components of the objective to “develop a contextual roadmap in each state”. Using purposive sampling, a director was chosen from each ministry of those who already participated in the earlier phases, preferably the director in charge of the programme. In addition the State Nutrition Officer who serves as the secretary of the State Committee for Food and Nutrition, was also included in the validation. The target was to recruit ten stakeholders per state. A total of nine (9) stakeholders (4 in Anambra State and 5 in Kebbi State) completed and returned the validation tool. The validation tool was self-administered, the participants were given the roadmap and the validation tool. The validation tool and the roadmap were e-mailed to all ten stakeholders with weekly reminders. After 1 month of initial emailing, two research assistants (one per state) also printed copies and delivered. The research assistants followed up with the stakeholders and collected the completed validation matrix. This was to improve participation and return. They graded using the Likert scale and commented on the acceptability, demand, implementation, translation, practicality, integration and potential buy-in of the developed roadmap.

### Data analysis

Data from all completed surveys were collated in a spreadsheet (Excel. Microsoft, 2016). Participant responses were checked for completeness for each of the validation tool. Negative responses implied the likelihood of the roadmap being misunderstood and unlikely to be adopted by government officials. Percentages were calculated for each of the items assessed for each domain or construct. A composite average score was then computed by adding itemised percentages per construct or domain. Data from the validation process was arranged in the tool and matched with the responses of each participant. Qualitative interpretation and synthesis of responses about feasibility, practical implementation, and the likelihood of political adoption of the roadmap were done manually via deductive analysis.

### Rigour and validity

Four criteria of trustworthiness helped ascertain rigour in this study [24, 25]. For credibility, experts used were provided with a detailed description of the study and thus had an excellent understanding of the research aims and context. For the validation, data collected was member-checked in a bid to allow the state stakeholders the opportunity to confirm the data. This was done during data analysis by the researcher who used telephone to communicate with the participants’ if required. For dependability, the research supervisor conducted verification exercises and ensured that the procedures followed were scientific and acceptable. For confirmability, completed questionnaires and transcripts are maintained for safekeeping and audit. For transferability, this has been achieved by providing a detailed description of the research, research setting and context.

In addition to the criteria of trustworthiness, the validity of the questionnaire was established in two ways. First through review by the research supervisors (experts in nutrition) and piloting with stakeholders.

### Research ethics

The study protocol was approved by the Health and Research Ethics Committee of Stellenbosch University (S13/09/171) and permission was obtained from all the ministries in Nigeria involved in the study. All participants further gave written consent after the study was explained to them in full. They were also given an option to withdraw from the study. The procedures adhered to the Declaration of Helsinki 2013 principles. This article focuses on the development and validation of the roadmap, the other outcomes have been reported elsewhere.

## Results

Results are presented under three sub-headings following the steps of the validation process.

### Expert review results

After the two rounds of the experts’ review, substantial changes were made on the roadmap. The changes from the expert review included changing of core concepts to more common words for clearer understanding. Secondly, a generic table for mainstreaming nutrition was added to the roadmap to provide a template easily adaptable by interested states. Finally, the experts introduced the utilization of nutrition indicators as monitoring aid for the states. Nutrition indicators such as low birth weight were thus added to the roadmap.

### Results of validation tool piloting

The responses to the pilot-testing were incorporated and used to refine the instrument to validate the roadmap. Changes were made to the validation instrument. These changes included the reframing of some validation questions and merging validation questions that were similar and appeared repetitive. Final revisions did not violate the earlier roadmap assumptions, and modifications after the pilot testing.

### Validation results

Of the stakeholders invited to participate, nine responded. All participants (9) showed an adequate understanding of the roadmap and other sections, as seen in the scores given in Table 3. Responses regarding the translation of the roadmap varied. The majority (86.6%) either strongly agreed or agreed that the result was translatable (43.0 and 43.6% respectively). Few editorial changes were made to the roadmap. Some stakeholder highlighted sentences that were unclear. The most significant change was strengthening the nutrition-sensitive action of agriculture. Table 3 below illustrates stakeholder validation percentages and verbatim responses.

**Table 3:** Qualitative and quantitative validation results (*n* = 9)

	Strongly agree (%)	Agree (%)	Neutral (%)	Disagree (%)	Strongly disagree (%)	Qualitative quotes
Understanding	43.8	43.0	13.20	–	–	*All agriculture related sections are clearly understood.*
Translation	43.0	43.6	–	13.4	–	*No additional cost can be seen as high for goal achievement.* *Budgetary allocation is usually grossly inadequate.*
Acceptability	51.3	38.6	–	10.3	–	*The roadmap stratifies and aligns to the ministry.*
Demand for a roadmap	26.3	73.7	–	–	–	*The roadmap address issues on goals, training, employment, and integration.*
Implementation	–	90.9		9.1	–	*Sections or recommendations are likely not be executed (institutional independence, develop indicators). (Staff) well trained to implement them (roadmap). The roadmap is well-articulated, and the programmes contained therein are capable of addressing the identified problem.* *(Implementable) with commitment and political will.*
Practicality	36.4	54.6	9.1	–	–	*No budgetary allocation or plan. The problem is how these will be sustained.* *The state has many problems to address therefore, choices will be made on which issues to address. This may take longer as funds are limited.*
Integration	37.5	62.5	–	–	–	*The roadmap can help the Ministry of Social Welfare to rise up to its challenges.*
Political buy-in	100	–	–	–	–	*Proposal should be sent to the stakeholders for a joint meeting to discuss the roadmap.*

### Understanding

About 13% of participants were neutral on their understanding of the roadmap. Specific sector comments include “*All agriculture-related sections are clearly understood*” as cited by participant 6.

### Translation

The section on translation sought to find out how appropriate the roadmap was for target audiences and adequate consideration of context. This also included any factor that would hinder the translation of roadmap to a working tool. While about 86% either agreed or strongly agreed on its translation, some stakeholders were sceptical on funding. Participant 2 stated that “Budgetary allocation is usually grossly inadequate”.

Others opined that translation had funding implications; it was worth it. *“No additional cost can be seen as high for goal achievement (stunting reduction)”* asserted participant 5.

### Acceptability

Major responses show that the roadmap was acceptable (about 51% strongly agreed). Acceptability was measured by appropriateness, fit for organizational culture, positive and negative effects on the organization. One stakeholder (participant 7) commented that: “*The roadmap satisfies and aligns to (with) the ministry*” where they worked.

### Demand

All stakeholder that responded to the demand questions either strongly agreed or agreed that there was demand for the roadmap. One stakeholder commented that: “*The roadmap address issues on goals, training, employment, and integration* “*(*Participant 3).

### Implementation

The stakeholders share the optimism that the roadmap is implementable and practical and will be successfully executed. All participants were willing to support the roadmap in their various ministries. These are some comments from the stakeholders:

*“Sections or recommendations are likely not be executed (institutional independence, develop indicators)”* (Participant 3)

*“(Staff) well trained to implement them (roadmap)”* (Participant 2)

*“The roadmap is well-articulated, and the programmes contained therein are capable of addressing the identified problem”* (Participant 6)

*“(Implementable) with commitment and political will”* (Participant 9)

### Practicability

Few stakeholders (9%) were neutral on how practicable the roadmap was. Most stakeholders again highlighted the unavailability of financial resources as a possible impediment for the roadmap implementation. Based on the frequency of stakeholders’ comments on budget allocation, it appears to be the most essential factor for successful implementation. In addition to finances, other concerns as shown in the comments below were sustainability and prioritization when the state is faced with numerous challenges. These are some of their comments:

*“No budgetary allocation or plan”* (Participant 4)

*“The problem is how these will be sustained”* (Participant 6)

*“The state has many problems to address therefore, choices will be made on which issues to address. This may take longer as funds are limited”* (Participant 8)

### Integration

Most stakeholders (63%) agree that the roadmap can be integrated into their existing leadership. One stakeholder commented that: “*The roadmap can help the Ministry of Social welfare to rise up to its challenges*” *(Participant 4).*

### Potential buy-in

Potential buy-in in this validation was strictly that of the senior government official responding to the validation questionnaire rather than the elected political office holders responsible for making decisions. All stakeholders were strongly optimistic about their support for the roadmap. Though a stakeholder suggested the need for further discussion.

*“Proposal should be sent to the stakeholders for a joint meeting to discuss the roadmap”* asserted participant 5.

### The roadmap for mainstreaming nutrition-sensitive interventions in Kebbi and Anambra states

Given the phenomenon of continued high but varied stunting rates across states in Nigeria, designing a targeted, practical and implementable suite of nutrition-sensitive interventions should be an utmost priority for state governments in Nigeria. An effective roadmap should be embedded in multiple domains that influence nutrition outcomes ensuring that efforts are complementary. The roadmap was developed and validated through a rigorous process. Additional file 1: Table S1 below shows the final version of state-specific roadmaps that should be implemented to ensure sustainable nutrition-sensitive mainstreaming in Anambra and Kebbi States, Nigeria.

## Study limitations

There was poor response for piloting of the validation tool and roadmap, instead of being administered to ten participants only two responded. This lead to the small sample size of pilot participants.

Secondly, the ideal main validation in Nigeria would have been a focus group discussion or workshop or roundtable discussion with all stakeholders present to help in gathering information on the dynamics of discussion on the roadmap. This was not feasible due to resource and time constraint. Thus, the instruments were self-administered. Thirdly, the absence of a participant from Anambra state’s validation exercise means that the perspectives of the ministry of education are missing from the operational validation.

For future studies, larger piloting and validation sample size is recommended, to ensure the reliability of the validation exercise. However, the foundation of the intervention remains solidly grounded on preceding phases’ empirical results.

## Discussion

Adapting contexts to programs can be challenging. To enhance the impact of nutrition-sensitive sectors, there is a need to develop a new roadmap adapted to local context that is implementable and provides real solutions on how these sectors can contribute to improving nutrition. This study aimed to complete the final development of the roadmap for mainstreaming nutrition-sensitivity in Nigerian states, incorporating expert reviews, piloting validation tool and applying it to determine operational validity of the developed roadmap. The utilisation of both the quantitative and qualitative methods described increased the data credibility, aiding the researchers in understanding the complexity of context-specific nutrition-sensitive mainstreaming in the states [20] and setting priorities that allow targeting through the established programme operations and pathway.

The purpose of validation is to explore if the developed contextual roadmap will function as intended once placed in the stakeholder’s environments and assess the roadmap’s likelihood for success in mainstreaming nutrition initiatives in the selected states. The importance of context to implementation of HIV/AIDS has been established using South Africa as an example [10]. Edwards and Barker [10] raise concerns that national programs might fail without consideration of context-sensitive designs. Contextual elements and lessons learnt from HIV/AIDS interventions include paying attention to cultural practices and gender norms, characteristics of the study population, characteristics of health facilities, characteristics of health workers and sources of funding, among others [10].

Trade-offs among the epidemiological, operational and political domains is to be expected going from MNIA framework [16]. Although the percentage of operational feasibility for all validation themes was considerably high and almost perfect in this study as depicted in Table 3. The participants might have been overly optimistic. Results might be due to enthusiasm about nutrition integration occurring in their sector. It is unrealistic to expect that all aspects of the roadmap would be successfully implementable despite being operationally implementable. Despite this knowledge, the roadmap has clearly not identified trade-offs. Trade-offs are not theoretical and will only be identified during implementation of the roadmap. Thus, it is expected that during implementation, some aspects or strategies of the roadmap in a given state would be stepped down for one with greater impact or one with at that given time more political support or even more likely to be funded or has a better fit into the organizational structure.

## Conclusion

The primary goal of this study was to validate a developed contextual roadmap for mainstreaming nutrition-sensitivity that addresses important elements of the contextualisation and targeting for two states in Nigeria. The validation process used the end-users of the roadmap who are supposed to be government stakeholders with the onus to reduce malnutrition in all its forms. These stakeholders were used in the development of the roadmap thus provides a structure for developing such holistic interventions and an opportunity for implementation of evidence-based contextual program modification. We recommend that end-users of any programme must be involved in the validation of such contextual programmes.

## Supplementary information

**Additional file 1: Table S1.** Roadmap for mainstreaming nutrition-sensitive interventions in Kebbi and Anambra States.

## Data Availability

Raw data, both print and electronic and the dissertation are being stored at the Division of Human Nutrition at Stellenbosch University. The data can be made available by request to the corresponding author.

## Abbreviations

ATASP: Agricultural Transformation Agenda Support Program 1
ECD: Early Childhood Development
IITA: International Institution of Tropical Agriculture
LGA: Local Government Area
MNIA: Mainstreaming Nutrition Initiative Assessment
NDHS: National Health Demographic Survey
PCOM-RAT: Political Commitment Rapid Assessment Tool
SAE: Small Area Estimation
UNICEF: United Nations Children’s Fund
WASH: Water, Sanitation and Hygiene
WHO: World Health Organisation
