# Supplementary material for: Validation of a roadmap for mainstreaming nutrition-sensitive interventions at state level in Nigeria
Source: Nutr J. 2020 Sep 9;19:96. doi: 10.1186/s12937-020-00612-1 (PMC7488070; doi:10.1186/s12937-020-00612-1)
Supplement: Supplementary file 1 — Additional file 1: Table S1. Roadmap for mainstreaming nutrition-sensitive interventions in Kebbi and Anambra States. [file 12937_2020_612_MOESM1_ESM.docx]

Table 1: Roadmap for mainstreaming nutrition-sensitive interventions in Kebbi and Anambra States

Roadmap for mainstreaming nutrition at state levels in Nigeria

- - - 1. **Introduction**

The long-term effect of the current malnutrition on human and state capital cannot be quantified. It is estimated that 37 percent of under-five children in Nigeria are stunted^[[1]](#footnote-1)^. The long-term effects of this on human and national development is quite profound, given the impact on education, productivity, and mortality. This document provides practical suggestions as to WHERE malnutrition efforts should be targeted, WHAT programmes should be targeted to promote nutrition-sensitivity, and HOW such programmes can be strengthened further. A study was conducted that led to the development of this roadmap (Ethics No: S17/05/099). This roadmap was designed to strengthen the ability of the state-level stakeholders to engage in and monitor nutrition-related interventions, policy development, and advocacy in the state. The roadmap is intended to facilitate the integration of nutrition-sensitive actions in various sectors. The roadmap consists of different sections and can be used separately or together in assisting stakeholders towards full nutrition-sensitive implementation. The roadmap includes an action plan for nutrition-sensitive actions (Roadmap Table 1), action plan for generating political commitment at the state level (Roadmap Table 2) and plans specifically for Kebbi and Anambra States (Roadmap Tables 3 and 4) respectively.

Roadmap Table 4.1: General Roadmap for mainstreaming nutrition-sensitive actions at the state level

| **Sector** | **Action** | **Critical success factors** | **Indicators** | **Sectoral input** | **Sectoral Output** | **Who** | **Outcome** |
| --- | --- | --- | --- | --- | --- | --- | --- |
| Health and nutrition | Targeting of vulnerable populations.  Integration of nutrition with other sectors.  Adequate implementation. | Funding.  Political commitment to nutrition.  Employing efficacious intervention.  Coverage of interventions.  Sustainability. | Low birth weight.  Underweight.  Increased morbidity.  Mortality rate. | Nutrition indicators and objectives in programmes.  Coverage.  Efficacious intervention. | Sustainability.  Project outcomes.  Improved nutritional status.  Improved women overall empowerment. | Ministries.  Directors.  Programme implementers. | **Improved nutritional status** |
| Education | Provision of quality ECD^[[2]](#footnote-2)^.  Produce quality ECD teachers.  Parents understanding and support of ECD education.  Awareness of ECD education and its significance. | Functional growth monitoring and tracking.  Funding.  Political commitment.  Monitoring and evaluation/quality control.  Formation of state and LGA^[[3]](#footnote-3)^ integrated ECD committee.  Child tracking system.  Alignment of Integrated ECD committee with SCFN. | Repetition rates in primary education.  Retention rates or grade achievements. | Quality teachers and caregivers.  Functional growth monitoring and tracking.  Cultural sensitivity. | Improved ECD quality.  Improved detection of growth faltering.  Increased enrolment. | State Ministry of Education. |  |
| Social welfare | Labour market-based skills acquisition training.  Nutrition education. | Targeting.  Integration.  Implementation.  Monitoring and evaluation/quality control. | Productivity losses from manual activities.  Productivity losses from non-manual activities. | Political commitment.  Skills acquisition training/curriculum.  Health and nutrition education components. | Improved skills.  Improved nutrition behaviours. | State Ministry of Social Welfare. |  |
| WASH | Develop healthy habitation.  Increased citizenry sanitation awareness. | Implementation.  Intersectoral collaboration.  Funding.  Political commitment.  Formation of state and LGA environmental task force.  Monitoring and evaluation/quality control. | Rate of diarrhoea.  Rate of soil-transmitted helminth infections.  Rate of environmental enteropathy. | Alignment of agencies and bodies.  Integrate Ministry of Environment into National Committee for Food and Nutrition, State Committee for Food and Nutrition and Local Government Committee for Food and Nutrition. | Improved habitation.  Increased citizens awareness of sanitation. | State Ministry of Environment.  National Committee of Food and Nutrition. |  |
| Agriculture | Rural infrastructure development.  Commodity value chain development. | Sustainability of programmes. | Women empowerment in agriculture.  Income from agriculture.  Own food production. | Integration.  Sustainability strategies.  Gender mainstreaming.  Nutrition Behavioural Change Communication. | Improved nutrition delivery through agriculture. | State Ministry of Agriculture. |  |

Roadmap Table 4.2: General Roadmap for generating political commitment in Nigerian states

| **Audiences** | **Activities** | **Indicators** | **Who** |
| --- | --- | --- | --- |
| Governors and Deputy governors. | - Invite them to a briefing on importance of nutrition. - Follow-up state champions on using their influence for malnutrition. | - Number of political commitment speeches. - Number of meeting held with nutrition audience. | - State Committee for Food and Nutrition - CSO^[[4]](#footnote-4)^. - Ministry of Health. |
| Commissioners of key nutrition-sensitive ministries. | - Track nutrition-sensitive budget. - Sector specific meeting on contribution to nutrition. | - Increased budgetary allocation to nutrition. - Percent of released funds. - Nutrition related activities in the line ministries. | - State Committee for Food and Nutrition Ministry of Budget and Economic Planning. |
| State Committee of Food and nutrition. | - Training needs assessment for the various line ministries on nutrition. - Nutrition budget strategy. | - Number of policy documents. - Alignment of ministerial programmes. | - Ministry of Health. |
| Civil Society Organisations. | - Map active CSOs in the state. | - Number of CSOs in the states. - Increase in number of Local Government Areas where CSOs work. | - Ministry of Health. - State Committee for Food and Nutrition |
| Media. | - Train state journalists on nutrition issues and nutrition reporting. - Follow up media houses on nutrition reporting in the state. - Regular press briefing on nutrition-related issues. | - Number of state-based media houses reporting on nutrition. - Media database. - Press briefing on nutrition. - Number of journalists trained on nutrition. | - State Committee for Food and Nutrition |

Roadmap Table 4.3: Roadmap for Anambra State

| Where | Goal | Action | Programme input | Programme output | Who |
| --- | --- | --- | --- | --- | --- |
| Education. | - Target identified LGAs. - Increase quality of Early Childhood Development. - Increase enrolment. | - Integration. - State level commitment. | - Employ more teachers. - Enhance quality assurance. - Improved child tracking system. - Training of parents. - Increased Funding. - Growth monitoring equipment and capacity. | - Improved enrolment and access to education. - Monitoring and evaluation reports. | - State directors. - Governor’s office. - SCFN^[[5]](#footnote-5)^. |
| Social Welfare. | - Improved skills^[[6]](#footnote-6)^ for employment. - Improved nutrition knowledge. | - Integration with other sectors. - Targeting of Local Government Areas with high burden. | - Improved training curriculum. - Availability of equipment^[[7]](#footnote-7)^. - Quality assurance. | - Improved skills acquisition. - Increased nutrition/health knowledge. | - State directors. - Governor’s office. - SCFN. |
| Environment. | - Improve sanitation in the state. - Improve citizen environmental awareness. | - Alignment of waste management and ministry. - Formation of state environmental task force. - Advocacy. - Intersectoral collaboration. - Develop indicators. - Institutional independence. | - Employ qualified environmental officers. - Implement environmental inspections. - Enhance quality assurance. - Active information and education campaigns and BCC^[[8]](#footnote-8)^. | - Monthly reports. - Improved sanitation. - Increased citizen awareness, knowledge and practice. | - State directors. - Governor’s office. - SCFN. |
| Agriculture. | - Increased farmers income. - Increased nutrition knowledge. | - Integration. - Sustainability strategies. | - Strengthen the nutrition BCC. - Train extension agents on nutrition pathways. - Strengthen exit strategies for sustainability. | - Increased farmers income. | - State directors. - Governor’s office. - SCFN. |

Roadmap Table 4.4: Roadmap for Kebbi State

| Where | Goal | Action | Programme input | Programme output | Who |
| --- | --- | --- | --- | --- | --- |
| Education. | - Target LGAs with high malnutrition. - Increase quality of ECD. - Increase enrolment. | - Integration. - State level commitment. - Cultural sensitivity. | - Employ more teachers. - Enhance quality assurance. - Improved child tracking system. - Training of parents. - Appropriate funding. - Growth monitoring equipment and capacity. | - Improved enrolment and access to education. - Monitoring and evaluation reports^[[9]](#footnote-9)^. | - State directors. - Governor’s office. - State Committee for Food and Nutrition |
| Social Welfare. | - Improved skills for employment. - Improved nutrition knowledge. | - Integration with other sectors. - Targeting of LGAs with a high burden. | - Improved training curriculum. - Availability of equipment. - Quality assurance of training programme. | - Improved skills acquisition. - Increased nutrition/health knowledge. | - State directors. - Governor’s office. - SCFN. |
| Environment. | - Improve sanitation in the state. - Improve citizen environmental awareness. | - Alignment of waste management and ministry. - Formation of state environmental task force. - Advocacy. - Intersectoral collaboration. - Develop indicators. - Cultural sensitivity in employment. | - Employ qualified environmental officers. - Implement environmental inspections. - Enhance quality assurance. - Active information and education campaigns and BCC^[[10]](#footnote-10)^. | - Monthly reports. - Improved sanitation. - Increased citizen awareness, knowledge and practice | - State directors. - Governor’s office. - State Committee for Food and Nutrition |
| Agriculture. | - Increased farmers income. - Increased nutrition knowledge. | - Integration. - Sustainability strategies. | - Strengthen the nutrition BCC. - Train extension agents on nutrition pathways. - Strengthen exit strategies for sustainability. | - Increased farmers income. | - State directors. - Governor’s office. - State Committee for Food and Nutrition |

1. NDHS 2013 [↑](#footnote-ref-1)
2. Early Childhood Development [↑](#footnote-ref-2)
3. Local Government Area [↑](#footnote-ref-3)
4. Civil Society Organisation [↑](#footnote-ref-4)
5. State Committee for Food and Nutrition [↑](#footnote-ref-5)
6. Refers to labour-based skills such as sewing, baking among others [↑](#footnote-ref-6)
7. Refers to equipment for skills acquisition [↑](#footnote-ref-7)
8. BBC - Behavioural Change Communication

   The LGAs with the highest burden of stunting in Anambra State, by percentage, is Anambra West (25.5 percent), Ayamelum (22.9 percent), Ogbaru (22.2 percent), Anambra East (20 percent), Awka North (19.9 percent), Onitsha North (19.8 percent), and Dunukofia (19.2 percent). The state stunting average is 19 percent. [↑](#footnote-ref-8)
9. This includes annual, biannual or midterm reports. [↑](#footnote-ref-9)
10. BCC - Behavioural Change Communication

    All LGAs’ stunting prevalence in Kebbi State are higher than the country average of 37 percent and 20 percent cut-off for public health problems. When categorised according to burden the local governments with the highest prevalence of malnutrition in Kebbi State include Maiyama (66.8 percent), Suru (66.5 percent), Kalgo (66.3 percent), Bunza (66.3 percent), Jega (65.4 percent), Wasagu/Danko (64.9 percent), and Gwandu (63.2 percent) . With the state stunting average being 63.5 percent, all LGAs in the state still need urgent action. [↑](#footnote-ref-10)
